# Observation of Mechanical and Kinetic Distinctions between Individual Isoleucine and Arginine Residues in a Peptide Dissociating from a Model Lipid Bilayer

**DOI:** 10.1021/acs.langmuir.5c05716

**Published:** 2026-07-15

**Authors:** Ryan S. Smith, Krishna P. Sigdel, Dylan R. Weaver, Stephen H. White, Martin B. Ulmschneider, Gavin M. King, Ioan Kosztin

**Affiliations:** ‡ Department of Physics & Astronomy, 14716 University of Missouri, Columbia, Missouri 65211, United States; § Department of Physics & Astronomy, 6647 California State Polytechnic University, Pomona, California 91768, United States; ∥ Department of Physics & Astronomy, 14747 University of Wisconsin—Eau Claire, Eau Claire, Wisconsin 54701, United States; ⊥ Department of Physiology and Biophysics, 8788 University of California, Irvine, Irvine, California 92697, United States; # Department of Chemistry, 4616 King’s College London, London SE1 1DB, United Kingdom; ∇ Department of Biochemistry, University of Missouri, Columbia, Missouri 65211, United States; ○ Materials Science and Engineering Instiute, University of Missouri, Columbia, Missouri 65211, United States

## Abstract

Peptide–lipid membrane interactions underlie many essential biological processes, yet the molecular determinants of peptide partitioning and dissociation from lipid bilayers remain incompletely understood. Here, we combine coarse-grained molecular dynamics (CG MD) simulations and atomic force microscopy (AFM)-based force spectroscopy to study the structural dynamics, energetics, and kinetics of penta-X5 peptides (WLLLX, with X = R or I) interacting with POPC bilayers. To elucidate how the position and identity of a single guest residue X modulates peptide–membrane interactions, we present these results in the context of the canonical Wimley–White penta-X (WLXLL) motif. Our findings from CG simulations are consistent with penta-X5 peptides adopting snorkeling conformations beneath the bilayer surface and with a dissociation scenario in which the final two or three residues detach almost simultaneously under the applied pulling force. Ensemble analyses of the reconstructed potential of mean force profiles lead to multiple energetic dissociation pathways. In combination with kinetic modeling of AFM rupture force distributions, the data reveal that both the mechanics (dissociation force) and kinetics (off rate) of peptide detachment are sensitive to the identity and sequence position of individual residues. These results highlight the power of integrating CG MD and single-molecule force spectroscopy to unravel residue-specific, sequence-dependent factors underlying peptide–lipid interactions.

## Graphical abstract

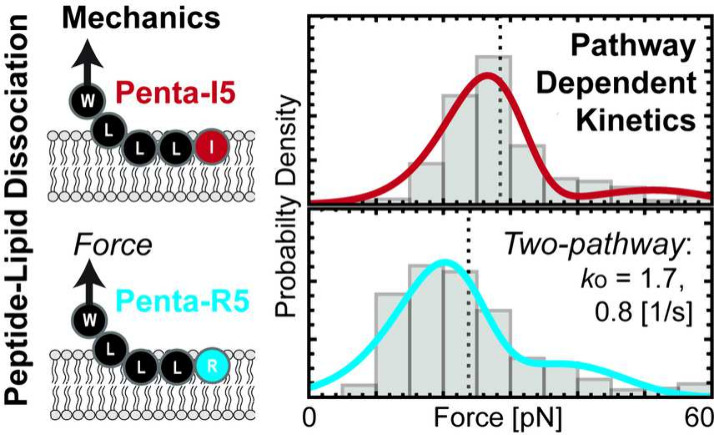

## Introduction

The interaction between short polypeptide segments and lipid membranes is fundamental to numerous biological processes, including antimicrobial activity, cell signaling, and membrane protein function. However, understanding the molecular determinants of peptide partitioning, insertion, and dissociation from lipid bilayers remains an important challenge in biophysical chemistry.^1^ Previous bulk thermodynamic studies by Wimley and White^2^ established the utility of model peptides like penta-X (WLXLL) in providing crucial insights into how the identity of a single guest residue (X) modulates peptide affinity with lipid bilayers.

While bulk experiments reveal ensemble-averaged thermodynamic properties, they cannot resolve contributions of individual residues at the single-molecule level nor can they capture the dynamic sequence of events during peptide–membrane association and dissociation.^3–7^ Computer simulations, in principle, offer residue-level resolution; however, all-atom molecular dynamics (MD) simulations are often impractical for these systems due to limited sampling of the relevant phase space and the computational cost associated with capturing rare events such as peptide dissociation. Coarse-grained (CG) MD simulations, combined with high-precision atomic force microscopy (AFM)-based force spectroscopy,^8^ provide a powerful approach for probing the structural dynamics, energetics, and kinetics of peptide–lipid interactions at the single-molecule level. CG models enable efficient exploration of configurational space, while AFM force spectroscopy directly measures the forces associated with peptide detachment from membranes in real time, yielding complementary insights into the underlying mechanisms.

In this study, we employ CG MD simulations and AFM-based force spectroscopy to investigate the interaction of penta-X5 peptides (WLLLX, where X = R or I) with POPC lipid bilayers. POPC was chosen as a neutral, single-component bilayer to provide a minimal and well-controlled membrane system for quantifying peptide–lipid interactions. Isoleucine (I) and arginine (R) were selected as guest residues as they represent two distinct cases of amino acid chemistry - strongly hydrophobic (nonpolar) and strongly hydrophilic (charged). This guest residue choice achieves high contrast in residue–membrane interactions while maintaining an unstructured and controlled peptide scaffold. The focus on the penta-X5 scaffold, as opposed to the canonical penta-X (WLXLL), with a centrally located X, is motivated by previous AFM experiments and CG simulations, which have suggested that, under a similar pulling geometry, dissociation of penta-L is dominated by the near-simultaneous detachment of the final two or three residues.^7^ Thus, while penta-X is well-suited for bulk thermodynamic measurements, where the guest residue X is effectively pinned beneath the membrane surface by the flanking L residues, in force spectroscopy experiments the influence of X is more pronounced when positioned at the force probe-distal end of the polypeptide chain, in this case the C terminus, where it directly affects the mechanics of dissociation. We compare penta-R5 and penta-I5 and show that both the dissociation force and dissociation rate of these short, unstructured peptides exhibit a measurable dependence on the individual residue type. The results provide insight into sequence-specific determinants of peptide–membrane interactions.

## Results and Discussion

### Membrane Penetration of Individual Residues

We first investigated the partitioning of penta-X and penta-X5 peptides into POPC lipid bilayers by conducting 5 μs free (i.e., unbiased) CG MD simulations performed without applied forces or restraints, excluding the initial 500 ns of equilibration from subsequent analyses. The distributions of residue penetration depths, Δ*z*_res_, into the POPC bilayer for (a) penta-X and (b) penta-X5 peptides are shown in Figure 1. Here, Δ*z*_res_ = *z*_res_ – *z*_P_, where *z*_res_ is the projection onto the membrane normal (the *z* axis) of the residue’s center-of-mass (COM) position vector and *z*_P_ denotes the position of the nearest lipid membrane surface. Although backbone bead positions provide an alternative reference that is less sensitive to side chain size, we use residue COM positions because they more directly characterize effective residue–membrane interactions relevant to AFM force spectroscopy. The peptide systems display broad, largely unimodal distributions, indicating that, although the peptides remain mobile within the bilayer, they exhibit a preference for certain conformations.

**Figure 1:**
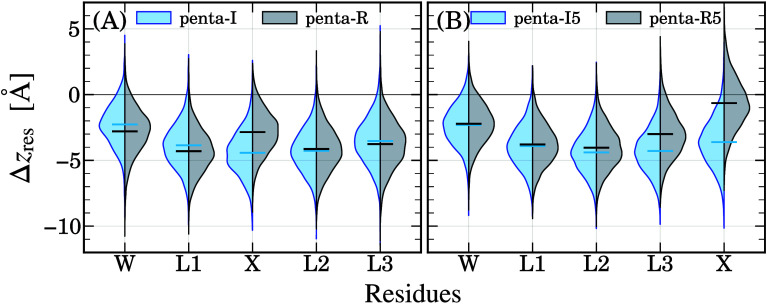
Membrane penetration depths in the absence of applied force. Depths are shown relative to the POPC bilayer surface, Δ*z*_res_, for individual residues in (A) penta-X and (B) penta-X5, as determined from coarse-grained molecular dynamics simulations. Semi-violin plots display the probability distributions for peptides containing guest residues X = I (blue) and R (gray). Horizontal bars indicate the mean penetration depth for each residue.

The mean conformations observed in POPC are consistent with previous findings using WL_*n*_ peptides (with *n* = 1, ..., 5, including penta-L or WL_4_),^7^ where W residues insert approximately 2 Å below the membrane surface and L residues typically position at *z*_res_ ≈ −4 Å. For peptides with guest residue X = I, the penetration profiles are similar across the polypeptide chain and are also similar to those of the penta-L peptide, suggesting a common mode of interaction among these hydrophobic residues.

In contrast, peptides incorporating X = R show distinct membrane insertion behaviors that depend on the position of the R residue. For penta-R5, with R at the C terminus, R snorkels up significantly and barely penetrates the bilayer surface (mean depth of <1 Å). In penta-R, where R is centrally located and surrounded by hydrophobic L residues, the mean depth of R is ∼3 Å below the membrane surface. These findings underscore the importance of both the identity and the position of the guest residue in shaping the peptide’s conformation within the bilayer. Such structural preferences are likely to impact both the dynamics observed during forced detachment and the underlying dissociation energetics.

### Peptide Dynamics During Forced Detachment

To characterize the conformational dynamics of the peptides during forced detachment, we performed a series of constant-velocity steered molecular dynamics (cv-SMD) and umbrella sampling (US) simulations. These simulations employed the reaction coordinate *z*_W_, which mimics the pulling geometry of the AFM tip during force spectroscopy, as detailed in the Experimental Section.

Figure 2 depicts the mean positions of each residue relative to the POPC bilayer center as a function of the reaction coordinate, *z*_W_, obtained from 52 independent simulations per system. Because L and I have similar hydrophobicity, the forced detachment pathways of penta-I5 and penta-I (Figure 2A and B) are similar to that of penta-L,^7^ and proceed in a largely sequential manner along the peptide backbone as W is retracted from the bilayer. In penta-I5, the terminal hydrophobic I residue remains closest to the membrane interface and detaches last together with the adjacent L_3_ residue, consistent with a peeling-like extraction pathway. In penta-I, the internal I residue detaches earlier, followed by the nearly simultaneous detachment of the terminal leucines L_2_–L_3_. In both cases, detachment is accompanied by modest local deformation, or puckering, of the bilayer surface, indicated by the dashed line in Figure 2; representative snapshots are shown in Figure S1.

**Figure 2:**
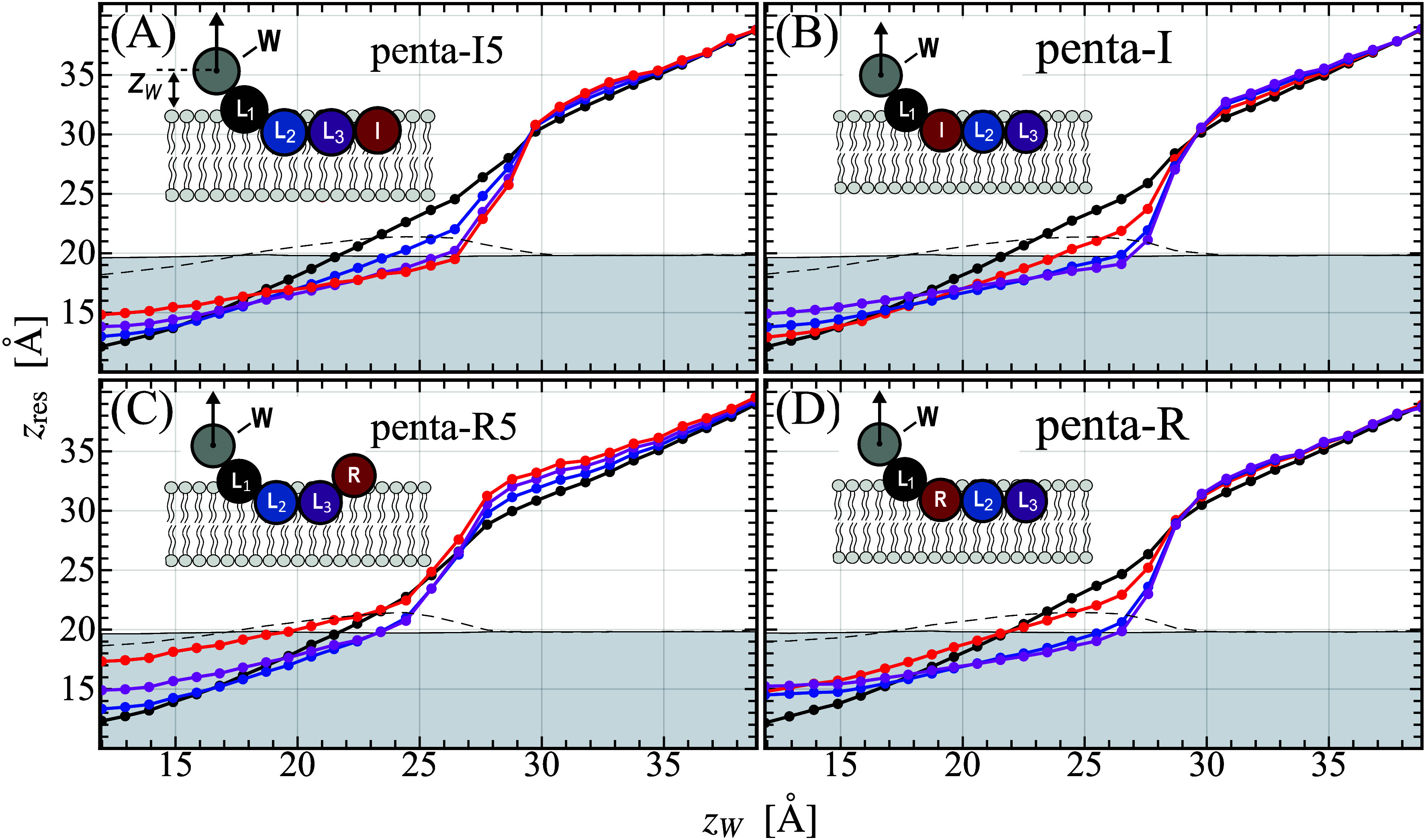
Simulations of AFM pulling experiments. Mean position measured from the center of the POPC bilayer, *z*_res_, of residues L_1_, L_2_, and L_3_ and guest residues I and R in peptides (A) penta-I5, (B) penta-I, (C) penta-R5, and (D) penta-R, plotted as a function of the mean position of residue W, *z*_W_, considered as reaction coordinate in the coarse-grained umbrella sampling simulations. Host leucine residues are color-coded consistently across all panels according to their order in the sequence, while guest residues are shown in red. The thin solid line represents the average phosphate surface, *z*_P_, of the corresponding POPC leaflet, and the gray region indicates the bilayer interior. The thin dashed curve shows the locally deformed membrane surface, defined as the mean *z*_P_ of phosphate beads within 12 Å of the peptide in the membrane plane.

In contrast, the hydrophilic arginine-containing peptides exhibit distinct detachment dynamics. For penta-R (Figure 2D), where the guest R residue is centrally positioned, the adjacent L residues are displaced slightly upward within the membrane when the peptide is pressed against or held near the bilayer surface in these SMD studies. Nevertheless, the residues detach sequentially along the peptide backbone: R exits after L_1_ and before L_2_ and L_3_. The detachment of penta-R is similar to that of penta-I, although here the charged R residue exits earlier than the hydrophobic I, likely because the hydrophilic character of arginine is partially screened by the surrounding L residues.

The behavior of penta-R5 (Figure 2C), with its terminal arginine, is qualitatively different from the previous cases. The terminal R residue remains approximately 3 Å closer to the membrane surface throughout the entire pulling process compared to the terminal hydrophobic residues of the other peptides. Consequently, it detaches from the bilayer at a *z*_W_ value 3–5 Å smaller than observed for those terminal residues, effectively disengaging from the membrane out of its expected sequential order. This early detachment is consistent with the charged R residue preferentially associating with the polar, locally deformed interfacial region rather than acting as a terminal hydrophobic anchor.

Across all simulations, the overall membrane integrity was preserved. The local bilayer surface deformed by at most 2–3 Å, and no lipid extraction, pore formation, or water permeation across the bilayer was observed. Representative snapshots of a dissociation event are provided in Figure S1. While the identity and sequence position of the guest residue modulate peptide interaction dynamics, these results support a dissociation scenario in which the final two to three force-probe–distal residues lose membrane contact over a narrow range of the reaction coordinate under the applied pulling force.

### Ensemble of PMFs

For both penta-X and penta-X5 peptides, an ensemble of *N*_s_ = 52 free energy profiles (PMFs), *U*(*z*_W_), was constructed from umbrella sampling simulations, as described in the Experimental Section. Each PMF exhibits a characteristic profile with a single free energy minimum (bound state) and a maximum (transition state), which are defined by an activation energy *U*_0_ (barrier height) and an activation length *x*_0_ (distance between the bound-state minimum and transition-state maximum along the reaction coordinate).^7,9^

The PMFs and corresponding distributions of *U*_0_ and *x*_0_ for penta-X peptides interacting with POPC are presented in Figure 3. These profiles are comparable to those previously determined for WL_*n*_ peptides,^7^ with minima located at *z* = *z*_W_ ≈ −2.5 Å. Notably, the PMFs and the distributions of *U*_0_ and *x*_0_ for penta-I and penta-R show similar mean values and overlapping distributions within statistical uncertainty. This observation is consistent with a dissociation scenario in which, under the applied pulling geometry, the dominant contribution to the rupture event arises from residues located at the force-probe–distal end of the peptide (see Figure 2). Consequently, the energetic contribution of the centrally located guest residue (X) is overshadowed by that of the final two leucine residues, effectively masking the significant physicochemical differences between isoleucine and arginine in the overall free energy profile.

**Figure 3:**
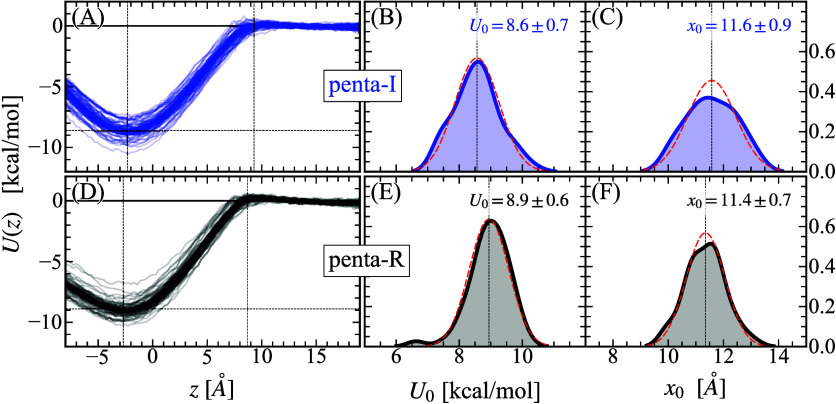
Potentials of mean force for penta-X. (A) Ensemble of PMFs (thin lines) and the mean PMF (thick line) for penta-I, shown as a function of the reaction coordinate *z* = *z*_W_, with corresponding distributions of (B) activation energy (*U*_0_) and (C) activation length (*x*_0_). (D–F) Corresponding plots for penta-R. Gaussian fits (red dashed lines) are overlaid on the distributions, with the mean and standard deviation values provided.

The corresponding results for the penta-X5 peptides, where the guest residue is positioned at the C terminus, are shown in Figure 4. In this series, the guest residue is expected to exert a more significant influence on dissociation energetics due to its location most distal to the attachment point of the peptide to the force probe (at the extreme C terminus). While the PMFs for penta-I5 closely resemble those of penta-I, the profile for penta-R5 reveals a distinct energetic landscape. Specifically, the mean activation energy, *U*_0_, for penta-R5 is approximately 2 kcal/mol lower than for penta-I5, as well as for penta-I and penta-R. This free energy difference is in overall agreement with scaled per residue transfer free energies, Δ*G*_X_, from the Wimley–White (WW) hydrophobicity scale.^2^ Indeed, using the WW values of free energies for transfer from lipid bilayer to water Δ*G*_R_ = −0.81 kcal/mol and Δ*G*_I_ = 0.31 kcal/mol, and applying the scaling factor of α ≈ 1.7 established in prior work,^7^ the resulting free energy difference is consistent with the value obtained from the present analysis.

**Figure 4:**
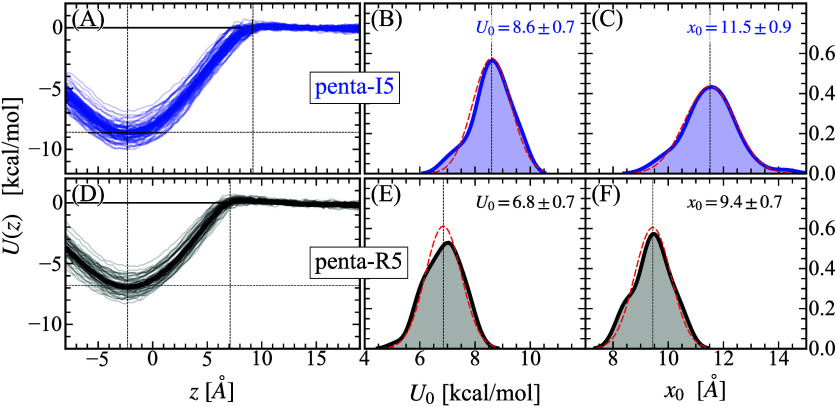
Potentials of Mean Force for penta-X5. (A) Ensemble of PMFs (thin lines) and the mean PMF (thick line) for penta-I5, with corresponding distributions of (B) activation energy and (C) activation length. (D–F) Corresponding plots for penta-R5. Gaussian fits (red dashed lines) are overlaid on the distributions, with the mean and standard deviation values provided.

The pronounced energetic difference between penta-I5 and penta-R5, in contrast to the similarity observed for the penta-X series, makes these penta-X5 peptides suitable candidates for detailed analysis via single-molecule AFM-based force spectroscopy measurements combined with theoretical modeling. To account for the stochastic nature of the dissociation process, we analyzed the ensemble of (*U*_0_, *x*_0_) pairs for the penta-X5 peptides, as shown in the scatter plot in Figure 5. The clustering analysis is used here solely as a statistical tool to identify distinct effective parameter sets (*U*_0_, *x*_0_) contributing to the dissociation-force distributions. These clusters do not imply distinct microscopic conformations or equally weighted populations, but rather represent alternative effective dissociation pathways within the one-dimensional stochastic escape framework. In this context, *U*_0_ and *x*_0_ are scalar descriptors of the effective PMF, *Ũ*(*x*): *U*_0_ is the barrier height and *x*_0_ is the distance between the bound-state minimum and transition-state maximum along the reaction coordinate. While a single-cluster analysis yields centroids (green) corresponding to the most probable values of the distributions, we employed *K*-means clustering to identify the two most predominant energetic pathways for dissociation. The centroids of these two clusters (red) define the mean PMF parameters for each distinct pathway, with the specific values provided in Table 1.

**Figure 5:**
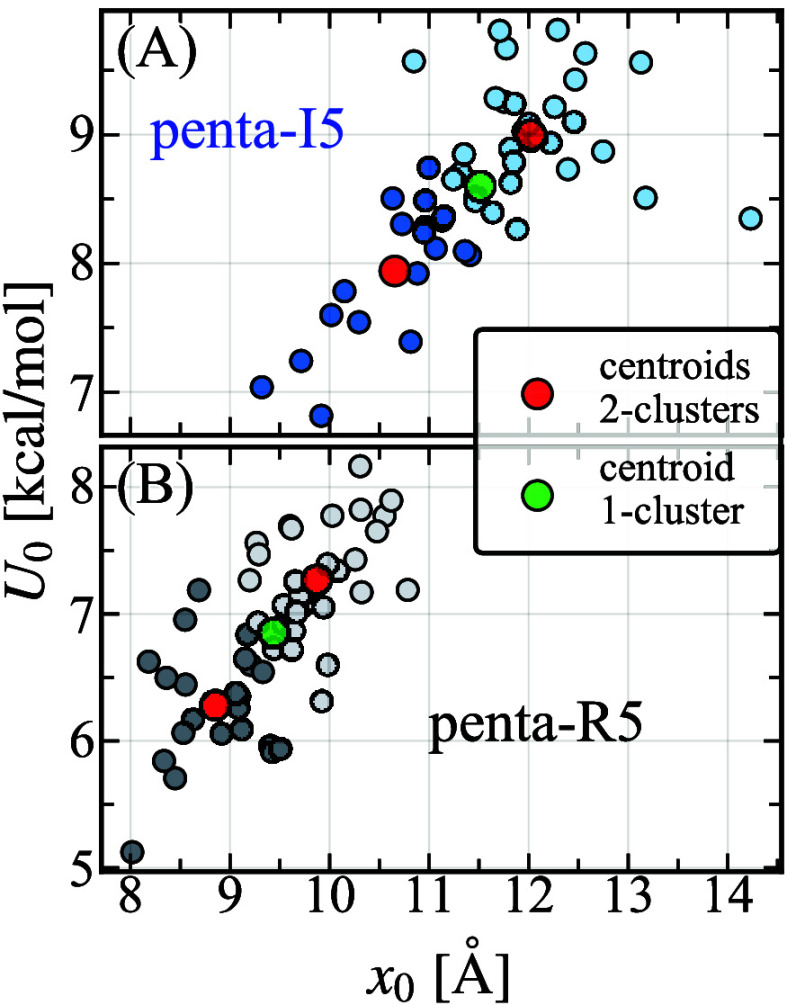
Scatter plot of (*x*_0_, *U*_0_) derived from 52 PMF profiles for (A) penta-I5 and (B) penta-R5. The centroid of all data points (single green circle) represents the mean PMF for a single, average energetic dissociation pathway. Alternatively, assuming two pathways, *K*-means clustering partitions the data into two groups (dark and light circles). The centroids of these clusters (two red circles) define the PMF for each distinct pathway. The values for *U*_0_ and *x*_0_ for both the single- and two-cluster cases are provided in Table 1.

**Table 1:** Mean Activation Energy (*U*_0_) and Characteristic Length (*x*_0_) with Standard Deviations for Penta-I5 and Penta-R5^a^

peptide	cluster	*U*_0_ (kcal/mol)	*x*_0_ (Å)
penta-I5	1 cluster	8.6 ± 0.7	11.5 ± 0.9
	2 clusters	9.0 ± 0.4	12.0 ± 0.7
		7.9 ± 0.5	10.7 ± 0.6
penta-R5	1 cluster	6.8 ± 0.7	9.4 ± 0.7
	2 clusters	7.3 ± 0.4	9.9 ± 0.4
		6.3 ± 0.5	8.9 ± 0.4

a Parameters were calculated using *K*-means clustering with assumptions of a single dissociation pathway (1 cluster) and two distinct dissociation pathways (2 clusters).

### Experimental Dissociation Force Distributions Combined with Theoretical Modeling

We sought to directly measure penta-X5 interactions with lipid bilayer membranes in physiologically relevant conditions. AFM-based force spectroscopy is a suitable technique for such measurements and has been employed to probe other polypeptide–lipid interactions.^3,5–7^ A cartoon of the experimental setup is shown (Figure 6A). Supported lipid bilayers were formed and tips were functionalized following established protocols (see the Experimental Section). In these experiments, the peptide was covalently attached to the AFM cantilever tip prior to bringing the tip into contact with the lipid bilayer. The flexible polyethylene glycol (PEG) linker allows multiple peptide binding orientations and does not interact with the bilayer, as verified via molecular dynamics simulations (see Figure S2 of the Supporting Information). After briefly (<1 s) and gently (≤200 pN) touching the functionalized AFM tip to the lipid surface, the piezoelectric stage affixed to the base of the cantilever was then retracted at a fixed speed *v* = 50 nm/s. The force curve (Figure 6B) shows a dissociation (rupture) event characterized by a sudden decrease in the measured force. Rupture force distributions, *P*(*F*), for penta-X5 peptides interacting with supported POPC bilayers were obtained from AFM measurements and analyzed using an established stochastic model^7,9–11^ described in the Experimental Section. Both the experimental and fitted theoretical *P*(*F*) curves are presented in Figure 6C–F. The histograms contain *N*_e_ = 388 rupture events collected from *N*_t_ = 11 distinct AFM tips for penta-R5; the corresponding values for penta-I5 are *N*_e_ = 895 and *N*_t_ = 8. The experimental data reveal a difference between the average force required to dissociate penta-I5 (28.7 pN) and penta-R5 (23.9 pN) (dotted lines in Figure 6C and E). Though small, this 4.8 pN difference is above the ∼1 pN noise floor of the AFM instrumentation employed here.^12^

**Figure 6:**
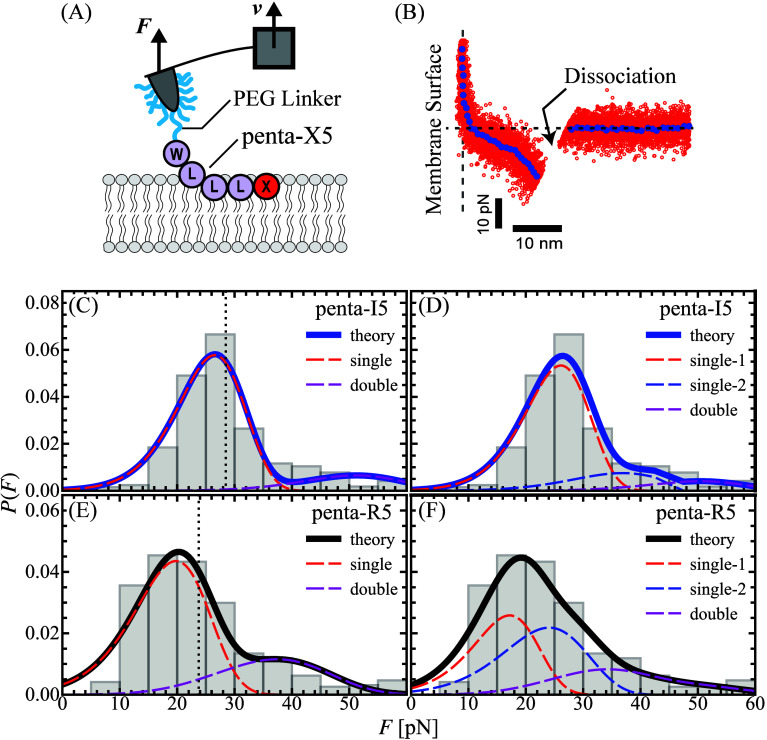
(A) Sketch of the AFM-based force spectroscopy experiment. (B) Force versus tip–sample separation trace showing a dissociation event for a penta-I5-functionalized tip dissociating from POPC. Full bandwidth (5 kHz, red) and smoothed (boxcar averaged to 10 Hz, blue) data are shown. Dissociation force distributions, *P*(*F*), for (C and D) penta-I5 and (E and F) penta-R5 in a POPC lipid bilayer. Mean values of the experimental distributions are shown as vertical dotted lines (C and E). Histograms are fitted to the theoretical model (thick solid lines). For both systems, the two-pathway dissociation model (D and F) provides a better fit to the data than the one-pathway model (C and E). The individual contributions of single- and double-rupture events for the two-pathway model are shown as thin dashed lines. The values of the *P*(*F*) model parameters are summarized in Tables 1 and 2.

Accurate modeling of the distributions required accounting for both single and double rupture events, particularly at forces exceeding 30 pN, consistent with the approach employed for WL_*n*_ peptides.^7^ The fitting analysis reveals that the intrinsic off rate, *k*_0_, for penta-R5 is approximately 1 order of magnitude higher than that of penta-I5, indicating that the hydrophobic I residue stabilizes the membrane-bound state. Conversely, substitution of the terminal I residue with a charged R significantly increases the peptide dissociation rate, thereby reducing its residence time at the bilayer interface. The complete fitting parameters and associated quality metrics are provided in Tables 1 and 2.

**Table 2:** Fitted Values of the Kinetic Model Parameters *k*_0_, the Intrinsic Dissociation Rate, and *w*, the Dissociation Pathway Probability (Weight), for the *P*(*F*) Dissociation Force Distributions of Penta-I5 and Penta-R5 in POPC Bilayers, under Single- (Single) and Two-Pathway (Single-1,2) Dissociation Models^a^

peptide	pathway	*k*_0_ (s^–1^)	*w*	τ (s)	χ^2^	BIC
penta-I5	single	0.17 ± 0.01	0.87 ± 0.01	5.9 ± 0.3	0.003	–1289
	single 1	0.17 ± 0.01	0.78 ± 0.03	5.9 ± 0.3	0.002	–1329
	single 2	0.06 ± 0.01	0.14 ± 0.03	16.7 ± 2.8		
penta-R5	single	1.43 ± 0.05	0.72 ± 0.01	0.7 ± 0.02	0.002	–1344
	single 1	1.67 ± 0.12	0.37 ± 0.04	0.6 ± 0.04	0.001	–1429
	single 2	0.83 ± 0.10	0.41 ± 0.03	1.2 ± 0.14		

a For the two-pathway model, “single-1” and “single-2” denote the two effective single-rupture components defined by the two *K*-means PMF centroids in Table 1. Mean dissociation time, τ (s), is given for each pathway. The lower chi-square (χ^2^) and Bayesian information criterion (BIC) values for the two-pathway model confirm its superiority over the single-pathway approach for both systems. Uncertainties represent the standard error of the mean (SEM).

Optimal fits for the penta-X5 systems were achieved by incorporating two distinct single rupture pathways, each characterized by different probabilistic weights *w* and intrinsic off rates *k*_0_. For both penta-R5 and penta-I5, these pathways exhibit distinct detachment kinetics, with one pathway displaying a lower dissociation rate and another a higher rate relative to the average single rupture behavior described above. Notably, the *k*_0_ of the “fast” dissociation pathway for penta-I5 remains four times slower than that of the “slow” pathway for penta-R5.

To further elucidate the contribution of multiple rupture events, theoretical *P*(*F*) distributions corresponding to idealized single rupture scenarios were computed and are shown in Figure 7. Removal of double rupture contributions from the fitted model produces notable changes in the resulting *P*(*F*) profiles. The effect is more pronounced for penta-R5, manifesting as a shift in the peak position, which reflects the approximately 2-fold higher weight of double rupture contributions for penta-R5 compared to penta-I5 in these experiments. These observations suggest that experimental conditions may favor certain dissociation pathways over others or facilitate simultaneous activation of multiple pathways rather than exclusive single-pathway dynamics.

**Figure 7:**
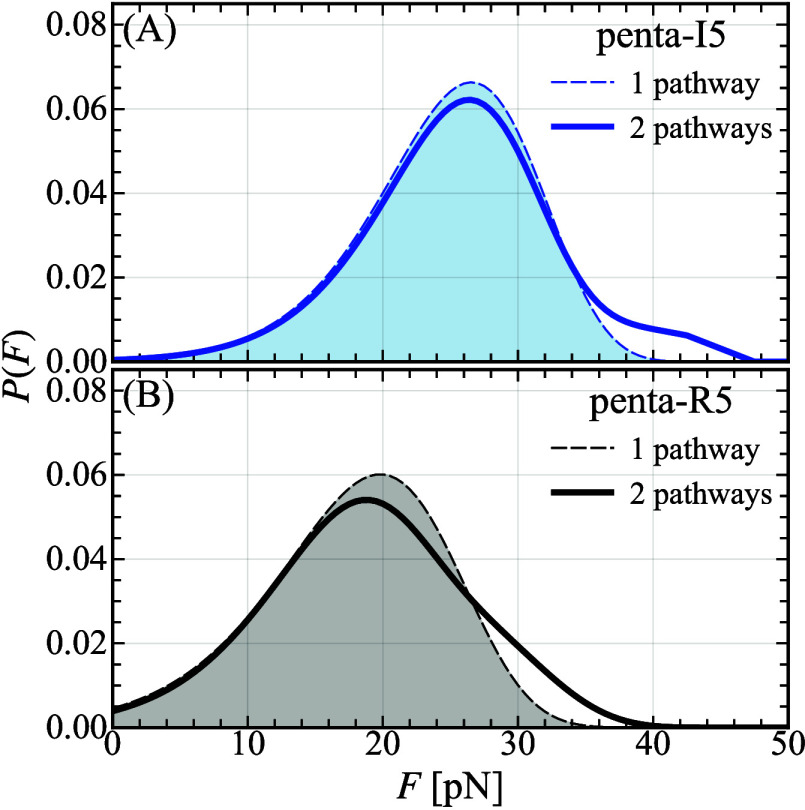
Predicted dissociation force distributions [*P*(*F*)] for (A) penta-I5 and (B) penta-R5 in a POPC bilayer, shown after removing the contribution of double-rupture events. Comparison of the one-pathway (dashed line) and two-pathway (solid line) models reveals a modest yet distinct difference between the two predictions.

## Conclusion

In this work, we resolve differences in dissociation forces and intrinsic off rates between a model hydrophobic and hydrophilic amino acid interacting with a supported lipid bilayer at the single-molecule level. A novel host–guest pentapeptide system that is well suited for single-molecule force spectroscopy of protein–lipid interactions is introduced. A fundamental aspect of such measurements is the propagation of tensile force along the molecular backbone. Oriented toward the force probe, this tension breaks a symmetry in the peptide–lipid interaction that limits the application of the conventional Wimley–White host–guest system. Our integrated AFM/simulation/analytical modeling approach enables comprehensive interpretation of the experimental dissociation force distribution, *P*(*F*), in terms of the dynamic energetic and kinetic landscape of the dissociation process.

Focusing on WLLLX peptides (X = I or R) in zwitterionic POPC bilayers, we used CG MD simulations to reconstruct the PMF profiles associated with peptide dissociation. Application of *K*-means clustering enabled the identification of predominant energetic dissociation pathways. By fitting the experimentally measured dissociation force distributions, *P*(*F*), to a theoretical model that incorporates the reconstructed PMFs as input, we calculated the dissociation (off) rates of penta-X5 from the membrane. Importantly, the methodology allows for deconvolution of the AFM data to extract the *P*(*F*) corresponding to a single peptide, independent of the number of peptides coupled to the AFM tip apex. Our findings further reveal that the pulling geometry leads to a dissociation scenario in which the final two or three residues lose membrane contact in quick succession. Importantly, both the mechanical (dissociation force) and kinetic (dissociation rate) properties of the short, unstructured peptides studied here show measurable sensitivity to the identity and sequence position of individual residues.

This study provides a quantitative framework for understanding how local sequence variations translate into measurable differences in peptide–membrane dissociation kinetics. By linking residue identity and sequence position to effective free-energy barriers and experimentally determined off rates, we show how mechanical loading geometry and sequence context together influence membrane residence times. Extending our previous study, which focused on the effect of polypeptide chain length, this work explicitly connects changes in single-residue identity to force-induced dissociation kinetics within a unified experimental-computational framework. We emphasize that the AFM force spectroscopy measurements and stochastic escape analysis employed here provide direct access to the intrinsic dissociation rate *k*_0_ (i.e., the off rate) governing peptide detachment from the membrane under mechanical load, but do not yield the association (on) rate *k*_on_ and therefore cannot be used to infer an equilibrium binding constant *K* = *k*_on_/*k*_off_. Nonetheless, this integrative strategy opens opportunities for dissecting the energetic and kinetic determinants of peptide–lipid interactions with the high scrutiny of single molecule technology, and may be applicable to the study of other biomolecules.

## Experimental Section

### Computer Modeling and Simulations

#### Building the Systems

All-atom models of the two pentapeptide variants were constructed using VMD:^13^ penta-X5 with the primary structure WLLLX and penta-X with the amino acid sequence WLXLL. Both peptides incorporated either hydrophobic (X = I) or hydrophilic (X = R) guest residues at the specified positions. The N and C termini were capped through acetylation and amidation, respectively, to maintain neutral charge states. Following solvation, each peptide system underwent energy minimization and equilibration protocols in *NVT* and *NPT* ensembles using NAMD 2.14 ^14^ with the CHARMM36 force field and TIP3P water model.^15–17^

Coarse-grained (CG) systems were subsequently prepared for each peptide using the CHARMM-GUI Martini Maker.^18–20^ The CG MARTINI representations^21^ of the peptides were positioned 14 Å above a fully hydrated 1-palmitoyl-2-oleoyl-*sn*-glycero-3-phosphocholine (POPC) bilayer patch. The CG models comprised 15 beads for penta-R and penta-R5 systems and 13 beads for penta-I and penta-I5 systems. The POPC bilayer contained 32 lipids per leaflet, with each side buffered by a 25 Å layer of 15 mM NaCl solution. The aqueous environment included approximately 1100 polarizable water molecules and 12 Na^+^ and Cl^–^ ions per side. To ensure charge neutrality in systems containing the positively charged arginine residue (X = R), an additional Cl^–^ ion was incorporated. The resulting CG systems contained approximately 4100 beads within periodic simulation cells with dimensions of 46 × 46 × 90 Å^3^. This system size was chosen as a compromise between computational efficiency and physical fidelity for short, local peptide–membrane interactions, enabling extensive ensemble sampling while maintaining stable bilayer properties. We emphasize, however, that larger bilayers would be required for larger peptides or for problems involving longer range membrane-mediated interactions.

#### Coarse-Grained (CG) MD Simulations

All CG MD simulations were performed with GROMACS 2021.2,^22^ using Martini 2.2 force-field parameters with polarizable water^21,23–26^ and periodic boundary conditions in the NPT ensemble. Constant pressure (*P* = 1 atm) and temperature (*T* = 300 K) were maintained using a semi-isotropic Berendsen barostat and velocity rescaling thermostat, with 5 and 1 ps coupling constants, respectively. A Verlet neighbor cutoff scheme was used. Short-range van der Waals interactions were truncated at 11 Å, and electrostatics were implemented using a reaction field with an 11 Å Coulomb cutoff. The integration time step was 20 fs. During simulations, both the area per lipid (APL), and the thickness (Δ*z*) of the POPC bilayer (defined as the distance between the planes of the phosphate P-beads in either leaflet) had generally accepted values,^27–31^ namely APL = 65 ± 1 Å^2^ and Δ*z* = 39 ± 1 Å. The bilayer center was defined as the midpoint between the mean *z* positions of the phosphate (P) beads of the two POPC leaflets, calculated independently for each simulation frame. The leaflet surface position, *z*_P_, was defined as the mean *z* position of the phosphate beads in the leaflet facing the peptide. Both quantities were recalculated dynamically for each frame.

Martini 2.2 is known to overestimate absolute peptide–lipid interaction strengths and does not provide a fully balanced description of protein–lipid interactions. In the present work, however, our interpretation relies primarily on relative comparisons between peptide variants with identical backbones, membrane composition, and pulling geometry, for which systematic force-field biases are expected to cancel to a significant extent. The consistency between the CG trends and the AFM measurements further supports the robustness of the qualitative conclusions. More quantitatively refined treatment of these systems using Martini 3 or atomistic simulations remains an important direction for future work.

Constant velocity steered molecular dynamics (cv-SMD) simulations were employed to guide the initial association of the peptide with the lipid membrane. In each system, the peptide was pulled toward the bilayer surface at a rate of *v* = 0.1 Å/ns using a harmonic biasing potential with a force constant of *k* = 5 kcal mol^–1^ Å^–2^. Following the initial cv-SMD simulation, each system underwent two consecutive 5 μs equilibration simulations with distinct roles. In the first equilibration stage, the backbone bead of the W residue was restrained to the plane defined by the lipid phosphate groups using a harmonic potential with *k* = 5 kcal mol^–1^ Å^–2^, allowing equilibration of peptide–lipid contacts while maintaining a well-defined reference position. From the final 4.5 μs of this restrained trajectory, *N*_s_ = 52 independent starting configurations were selected for each system, ensuring a uniform spatial distribution of the W residue across the membrane plane.

In the second equilibration stage, all restraints were removed and a fully unbiased (“free”) 5 μs CG MD simulation was performed. This trajectory was used to characterize equilibrium peptide partitioning and residue penetration depths reported in the Membrane penetration section and Figure 1. The selected configurations from the first (restrained) equilibration stage were used as initial states for an equal number of CG cv-SMD simulations to sample the reaction coordinate *z*_W_, defined as the distance between the W residue and the bilayer phosphate plane. The reaction coordinate was sampled over the range *z*_W_ ∈ [−8, 19] Å using the same steering velocity and force constant.

#### Umbrella Sampling and PMF Calculations

The potential of mean force (PMF) profiles for the penta-X and penta-X5 systems were constructed as a function of the reaction coordinate *z*_W_, defined as the distance between the tryptophan (W) backbone bead and the bilayer surface (the plane of the phosphate beads). These profiles were generated using coarse-grained (CG) umbrella sampling (US)^32^ in conjunction with the weighted histogram analysis method (WHAM).^33^ For each PMF calculation, a total of *N*_w_ = 28 US windows were established with centers separated by 1 Å, spanning a range from *z*_W_ = −8 to 19 Å. A harmonic biasing potential with a spring constant of *k* = 5 kcal mol^–1^ Å^–2^ was applied in all US simulations. Adequate overlap between adjacent umbrella sampling windows was verified during WHAM analysis, and PMF convergence was assessed through reproducibility across ensembles of independent calculations.

To enhance sampling and statistical robustness, *N*_s_ = 52 independent PMFs were generated for each peptide system, initiating each calculation from a unique starting configuration extracted from the corresponding cv-SMD trajectories. Within each of these *N*_s_ sets, every umbrella window was simulated for 40 ns. Finally, the mean PMF for each system was determined by averaging the resulting ensemble of *N*_s_ = 52 individual PMF profiles.

### Theoretical Modeling of *P*(*F*)

The dissociation force distribution, *P*(*F*), for a single peptide detaching from the bilayer was modeled based on the theory of stochastic escape over a free energy barrier.^34–38^ The distribution is given by^9–11^

1 $$P\left(F\right)=\frac{k\left(F\right)}{Ḟ}\exp\left[-\int_{0}^{F}\frac{k\left(f\right)}{Ḟ}df\right]$$

where the force-dependent dissociation rate, *k*(*F*), is derived from the potential of mean force (PMF):^9^

2 $$k\left(F\right)=k_{0}\frac{\int_{x_{-}}^{x_{+}}dye^{u\left(y,0\right)}\int_{-\infty }^{y}dze^{-u\left(z,0\right)}}{\int_{x_{-}}^{x_{+}}dye^{u\left(y,F\right)}\int_{-\infty }^{y}dze^{-u\left(z,F\right)}}$$

In these equations, *Ḟ* = *k*_s_ *v* is the force loading rate (cantilever stiffness *k*_s_ ≈ 12 pN/nm, retraction speed *v* = 50 nm/s), *k*_0_ is the intrinsic dissociation rate, *Ũ*(*x*) is the mean PMF obtained from CG US simulations, and *u*(*x*, *F*) = (*Ũ*(*x*) – *Fx*)/*k*_B_ *T* is the dimensionless potential under an applied force *F*. The integration limits *x*_–_ and *x*_+_ represent the reaction coordinate values at the bound state minimum and transition state maximum of the PMF, respectively.

Because the AFM tip may be functionalized with more than one peptide, two independently bound peptides can detach within the finite temporal resolution of the measurement and appear as a single rupture event; we refer to such events as double ruptures. Accordingly, the measured force distribution, *P*_exp_(*F*), was modeled as a linear combination of single-rupture events, *P*(*F*), and double-rupture events, *P*_d_(*F*).^7,9,11^ The double-rupture distribution, *P*_d_(*F*), was calculated as the self-convolution of the single-rupture distribution. For the one-pathway analysis, this composite model was fitted using one intrinsic dissociation rate *k*_0_ and one single-rupture weight *w*. For the two-pathway analysis, the same framework was extended by using the two *K*-means centroids in Table 1 to define two effective single-rupture PMF inputs, each with its own fitted *k*_0_ and weight *w* as reported in Table 2; the remaining probability accounts for double-rupture events.

### AFM Force Spectroscopy Experiments

#### Supported Lipid Bilayer Preparation

POPC lipid was purchased (Avanti Polar Lipid) in chloroform, blown dry with Ar, and placed in a vacuum chamber overnight. A dry mechanical vacuum pump (XDS5, Edwards) was used to prevent back streaming of oil, a potential contaminant. Dried lipids were suspended in 100 mM Na_3_PO_4_, pH 7.6. Liposomes were prepared by extrusion through a membrane (∼25 times, 100 nm pore diameter). Supported lipid bilayers were formed by vesicle fusion (1 mM, 45 min incubation, ∼30 °C) to clean glass surfaces, as described previously.^39,40^

#### Force Spectroscopy

For force spectroscopy, AFM cantilevers were functionalized following established protocols with minor modifications.^6,41,42^ To increase force precision, cantilevers with reflective coating removed (Biolever, BL-RC150VB-HW, Olympus) were employed.^12^ Spring constants were in the range 4–10 pN/nm, as determined via thermal calibration.^43,44^ Peptides were synthesized (Peptide 2.0) at 98% purity with the following sequences: penta-R5 = C–W–L–L–L–R and penta-I5 = C–W–L–L–L–I. The cysteine residue at the N terminus of each construct allowed site-specific, covalent binding onto AFM tips via a 9.5 nm long NHS-PEG_24_-maleimide linker (Thermo Scientific). Cantilevers were oxygen plasma cleaned (10 min, 30 W, Harrick Plasma), immersed in silane (3-ethoxydimethylsilyl)­propylamine (Sigma-Aldrich) for 60 s, and baked at 80 °C for 30 min. Tips were incubated in sodium borate (50 mM, pH 8.5) for 1 h, followed by the NHS-PEG_24_-maleimide linker for 1 h, and then peptide at 100 μM for 2 h. Finally, tips were washed (10 mM HEPES, pH 7.2, 75 mM Na_3_PO_4_) and loaded into the microscope for force spectroscopy experiments which were carried out in aqueous buffer solution (100 mM Na_3_PO_4_, pH 7.6) at ∼25 °C using a custom-built AFM instrument, described elsewhere,^45,46^ with tip retraction speed *v* = 50 nm/s. To minimize artifacts, the compressive force applied to the lipid bilayer was ≤200 pN and events occurring <3 nm above the lipid surface were excluded from analysis. Dissociation events exhibiting rupture forces >60 pN were rare and excluded from analysis. After applying these criteria, the remaining rupture events yielded consistent force distributions across independent AFM tips and experiments, as expected for specific peptide–lipid interactions.

## Supplementary Material



## Notes

Representative CG simulation snapshots illustrating peptide dissociation from a POPC bilayer (Figure S1) and molecular dynamics simulation of the PEG linker with the POPC bilayer (Figure S2) (PDF)

## Notes

*In Memoriam*: This manuscript is dedicated to the memory of Stephen H. White.
