# Supplementary material for: Observation of Mechanical and Kinetic Distinctions between Individual Isoleucine and Arginine Residues in a Peptide Dissociating from a Model Lipid Bilayer
Source: Langmuir. 2026 Jul 15;42(29):20888–97. doi: 10.1021/acs.langmuir.5c05716 (PMC13421991; doi:10.1021/acs.langmuir.5c05716)
Supplement: Supplementary file 1 [file la5c05716_si_001.pdf]

Supporting Information

Observation of mechanical and kinetic distinctions between individual isoleucine and arginine residues in a peptide dissociating from a model lipid bilayer

Ryan S. Smith, Krishna P. Sigdel, Dylan R. Weaver, Stephen H. White, Martin B. Ulmschneider, Gavin M. King*, and Ioan Kosztin*

Correspondence to: kinggm@missouri.edu; kosztini@missouri.edu

Table of Contents

Figure S1: Representative CG simulation snapshots illustrating peptide dissociation from a POPC bilayer

Figure S2: Molecular dynamics simulation of the PEG linker with the POPC bilayer

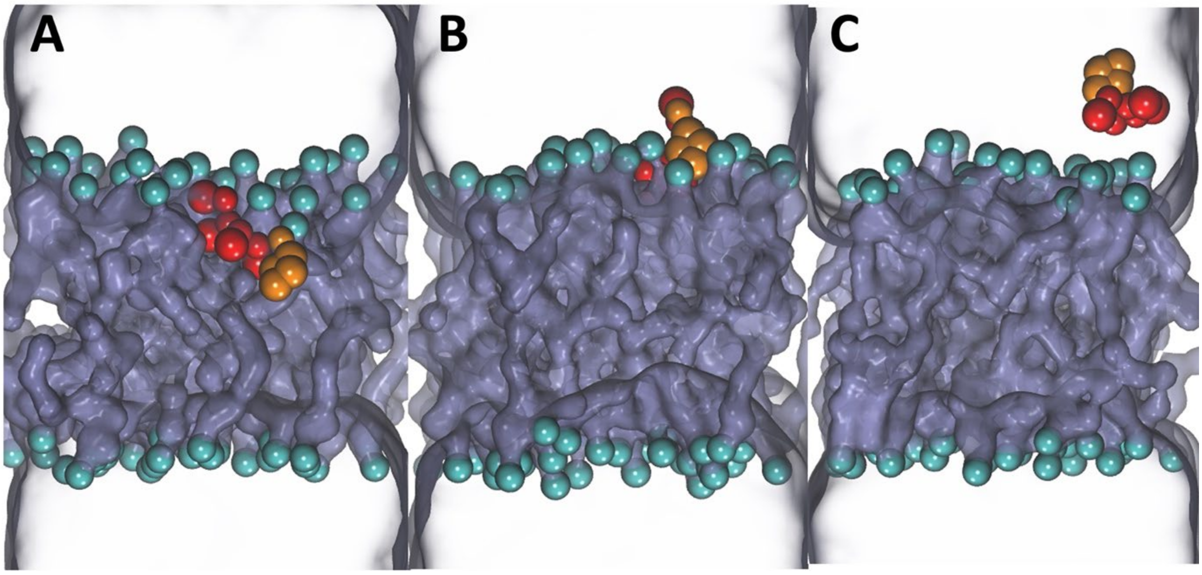

Figure S1. **Representative CG simulation snapshots illustrating peptide dissociation from a POPC bilayer**. (**A**) bound state, (**B**) near the transition state, and (**C**) fully dissociated state. The membrane remains intact throughout the pulling process, showing only localized surface deformation, with no lipid extraction or water permeation. Snapshots were rendered in VMD. Water beads are shown as a transparent silver surface, using an increased radius scaling and a reduced density isovalue to improve visual continuity near periodic boundaries. Lipid beads are depicted as translucent dark-blue surfaces. The pentapeptide is shown as opaque red spheres, with the N-terminal Trp highlighted in orange. Lipid phosphate beads are represented as opaque cyan spheres.

Molecular dynamics simulation of the PEG linker with the POPC bilayer

To investigate the molecular interactions of the PEG linker attached to the cantilever tip with the POPC bilayer we constructed an atomic detail model, exactly replicating the linker chemistry. The linker was then inserted spanning the membrane and after equilibration we performed a 1 μs unbiased equilibrium simulation. **Figure S2** shows that the linker exits the membrane after about 250 ns and plotting the center of mass motion shows that it freely samples the aqueous phase without binding to the membrane. This demonstrates that the linker chemistry is sufficiently hydrophilic to not bias the binding of any attached peptide to the membrane. Simulations were performed and analyzed with gromacs 4.6 (www.gromacs.org) and hippo beta (www.biowerkzeug.com), using the OPLS all-atom protein force field [1] in conjunction with the TIP3P water model [2]. OPLS united atom lipid parameters for POPC were taken from Ulmschneider et al. [3]. Electrostatic interactions were computed using PME,

and a cutoff of 10 Å was used for van der Waals interactions. Bonds involving hydrogen atoms were constrained using LINCS [4]. The integration time-step was 2 fs and neighbor lists were updated every 5 steps. All simulations were performed in the NPT ensemble, without any restraints or biasing potentials. Water, ions (100 mM NaCl), lipids, and the protein were each coupled separately to a heat bath with time constant τ_T = 0.1 ps using velocity-rescaling temperature coupling [5]. Atmospheric pressure of 1 bar was maintained [6] using weak semi-isotropic pressure coupling with compressibility $\kappa_z = \kappa_{xy} = 4.6 \cdot 10^{-5}$ bar^{-1} and time constant τ_P = 1 ps.

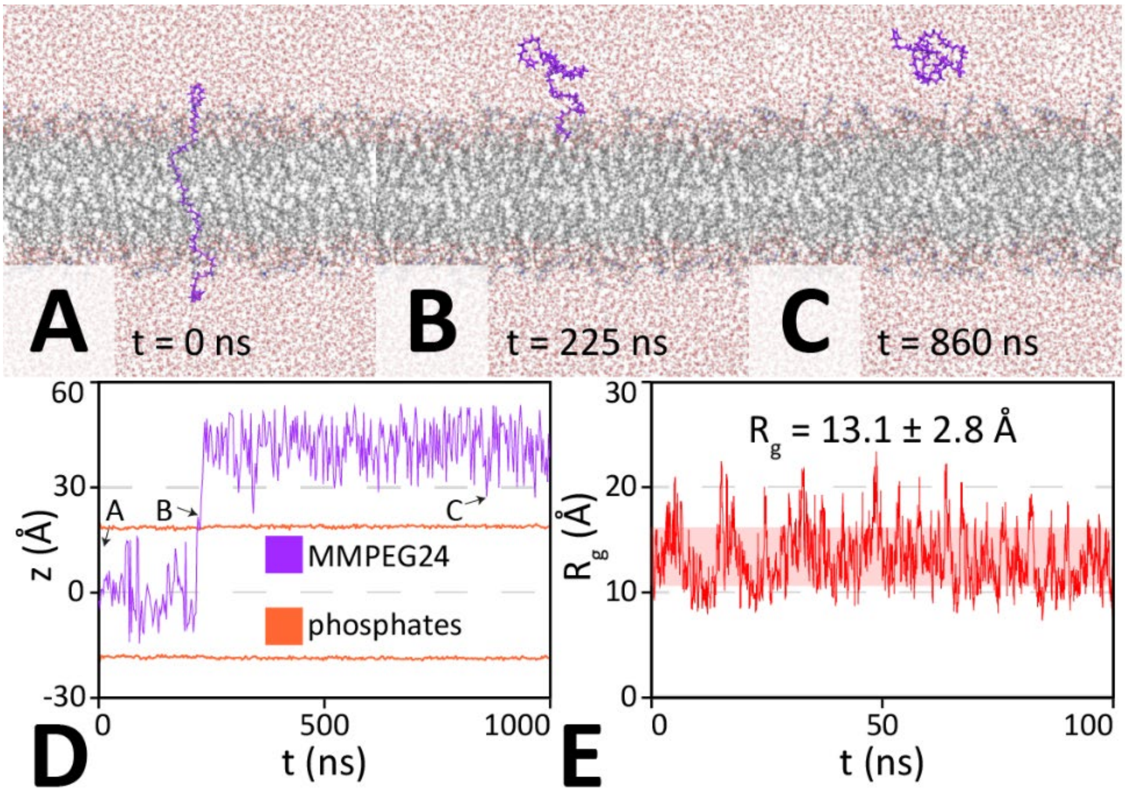

Figure S2. Molecular dynamics simulation of the PEG linker with the POPC bilayer. A-C. Snapshots from an unbiased 1 μs simulation show that the linker, initially inserted to span the membrane, exits the bilayer within ~250 ns and does not reattach to the membrane again. **D.** Center of mass position of the MMPEG24 linker with respect to the membrane normal (purple). The averaged phosphate positions for each membrane leaflet are shown in orange. **E.** Radius of gyration calculated from a 100 ns simulation of the MMPEG24 linker in a cubic box of water after equilibration for 50 ns. A reddish rectangle is centered at the mean Rg with height giving +/- the standard deviation, as reported on the chart itself.

References Cited

1. W. L. Jorgensen, D. S. Maxwell and J. Tirado-Rives, Development and testing of the OPLS all-atom force field on conformational energetics and properties of organic liquids, J..Am.Chem.Soc. **118**, 11225–11236 (1996).

2. W. L. Jorgensen, J. Chandrasekhar, J. D. Madura, R. W. Impey and M. L. Klein, Comparison of simple potential functions for simulating liquid water, J.Chem.Phys. **79** (2), 926–935 (1983).

3. J. P. Ulmschneider and M. B. Ulmschneider, United atom lipid parameters for combination with the optimized potentials for liquid simulations all-atom force fields, Journal of Chemical Theory and Computation **5**, 1803–1813 (2009).

4. B. Hess, H. Bekker, H. J. C. Berendsen and J. G. E. M. Fraaije, LINCS: A linear constraint solver for molecular simulations, Journal of Computational Biology **18**, 1463–1472 (1997).

5. G. Bussi, D. Donadio and M. Parrinello, Canonical sampling through velocity rescaling, The Journal of Chemical Physics **126**, 014101 (2007).

6. H. J. C. Berendsen, J. P. M. Postma, W. F. van Gunsteren, A. DiNola and J. R. Haak, Molecular dynamics with coupling to an external bath, J.Chem.Phys. **81** (8), 3684–3690 (1984).
